# Evolution of Echocardiographic Abnormalities Identified in Previously Healthy Individuals Recovering from COVID-19

**DOI:** 10.3390/jpm12010046

**Published:** 2022-01-04

**Authors:** Cristina Tudoran, Mariana Tudoran, Talida Georgiana Cut, Voichita Elena Lazureanu, Cristian Oancea, Adelina Raluca Marinescu, Silvius Alexandru Pescariu, Gheorghe Nicusor Pop, Felix Bende

**Affiliations:** 1 Department VII, Internal Medicine II, University of Medicine and Pharmacy “Victor Babes” Timisoara, E. Murgu Square, Nr. 2, 300041 Timisoara, Romania; tudoran.cristina@umft.ro (C.T.); tudoran.mariana@umft.ro (M.T.); bende.felix@umft.ro (F.B.); 2 Center of Molecular Research in Nephrology and Vascular Disease, Faculty of Medicine, University of Medicine and Pharmacy “Victor Babes” Timisoara, E. Murgu Square, Nr. 2, 300041 Timisoara, Romania; 3 County Emergency Hospital, Liviu Rebreanu Str., Nr. 156, 300041 Timisoara, Romania; 4 Department XIII, Discipline of Infectious Diseases, University of Medicine and Pharmacy “Victor Babes” Timisoara, E. Murgu Square, Nr. 2, 300041 Timisoara, Romania; lazureanu.voichita@umft.ro (V.E.L.); oancea@umft.ro (C.O.); adelina.marinescu@umft.ro (A.R.M.); 5 Doctoral School, Victor Baes university of Medicine and Pharmacy, 300041 Timisoara, Romania; pescariu.alexandru@umft.ro; 6 Department VI, Cardiology, University of Medicine and Pharmacy “Victor Babes” Timisoara, E. Murgu Square, Nr. 2, 300041 Timisoara, Romania; pop.nicusor@umft.ro; 7 Center of Advanced Research in Gastroenterology and Hepatology, Faculty of Medicine, University of Medicine and Pharmacy “Victor Babes” Timisoara, 300041 Timisoara, Romania

**Keywords:** COVID-19, transthoracic echocardiography, left ventricular function, diastolic dysfunction, systolic pulmonary artery pressure, right ventricular dysfunction, pericardial effusion/thickening

## Abstract

(1) Background: Although the infection with the SARS-CoV-2 virus affects primarily the lungs, it is well known that associated cardiovascular (CV) complications are important contributors to the increased morbidity and mortality of COVID-19. Thus, in some situations, their diagnosis is overlooked, and during recovery, some patients continue to have symptoms enclosed now in the post-acute COVID-19 syndrome. (2) Methods: In 102 patients, under 55 years old, and without a history of CV diseases, all diagnosed with post-acute COVID-19 syndrome, we assessed by transthoracic echocardiography (TTE) four patterns of abnormalities frequently overlapping each other. Their evolution was followed at 3 and 6 months. (3) Results: In 35 subjects, we assessed impaired left ventricular function (LVF), in 51 increased systolic pulmonary artery pressure, in 66 diastolic dysfunction (DD) with normal LVF, and in 23 pericardial effusion/thickening. All TTE alterations alleviated during the follow-up, the best evolution being observed in patients with pericarditis, and a considerably worse one in those with DD, thus with a reduction in severity (4) Conclusions: In patients with post-acute COVID-19 syndrome, several cardiac abnormalities may be assessed by TTE, most of them alleviating in time. Some of them, especially DD, may persist, raising the presumption of chronic alterations.

## 1. Introduction

As the infection with the SARS-CoV-2 virus has evolved into the most significant pandemic in recent centuries, representing a huge burden for health systems worldwide, over 250 million people became infected and more than 5 million died. Although respiratory manifestations prevail, multi-systemic injuries are common, among which the cardiovascular (CV) ones are responsible for the increased morbidity and mortality related to this disease [1,2,3]. The principal pathophysiological mechanisms considered responsible for the myocardial damage caused by COVID-19 infection are direct virus-induced injury and subsequent alterations due to the cytokine storm and inflammatory responses [4,5,6,7].

Several attempts to classify CV complications have been made, such as in primary involvements, including arrhythmias, acute coronary syndrome, myocarditis, thrombotic events, and arterial hypertension. Secondary CV involvements were considered interstitial cardiac fibrosis (ICF), cardiomyopathies, chronic heart failure (CHF), and postural orthostatic hypotension (POH), explaining, at least partially, the persistence of symptoms in post-acute and long COVID-19 syndromes [8]. Of course, there is an overlap between these forms, not to mention that an increased percentage of individuals infected with the SARS-CoV-2 virus have serious pre-existing CV diseases that are associated with a worse prognosis of COVID-19. Another contributing factor is the important CV side effects of new therapies (antivirals, antimalarials, and corticoids) used to treat this illness [9].

The real prevalence of CV complications is unclear and their diagnosis is often overlooked during the acute illness, taking into account that symptoms such as dyspnea, fatigue, and palpitations are attributed to pulmonary injury and hypoxia. Furthermore, some more subtle symptoms and clinical signs can be overlooked due to the immense burden put on health care workers who are overwhelmed by the enormous number of infected persons [8,10]. Thus, some non-invasive and accessible diagnostic methods such as electrocardiography (ECG) and especially transthoracic echocardiography (TTE) offer the possibility of identifying most of the alterations of the cardiac morphology and function, allowing us more accurate diagnosis and a follow-up during recovery [11].

Since COVID-19 is a relatively recent illness, the course of CV complications is still unclear. It is speculated that most of complications resolve sooner or later after the acute illness, but exact data are lacking so that further studies are needed to clarify this aspect.

This study aimed to follow the evolution of several abnormalities such as alterations of left ventricular function (LVF), diastolic dysfunction (DD), elevated systolic pulmonary artery pressure (sPAP), right ventricular dysfunction (RVD), or pericardial effusion (PE) or thickening (PT) diagnosed by TTE after 4 to 8 weeks from the acute illness in apparently healthy individuals who were recovering from COVID-19 but still presented remaining symptoms, which classified them as suffering from post-acute COVID-19 syndrome. Another aim was to observe which factors could influence the evolution of these pathological findings among patients infected with the SARS-CoV-2 virus during the first three waves of this infection.

## 2. Materials and Methods

### 2.1. Study Population

**Figure 1:**
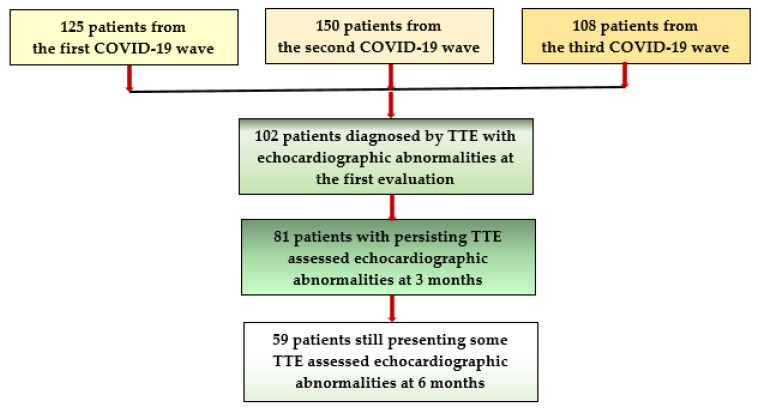
Flowchart of patient selection and follow-up.

Of all individuals who suffered from COVID-19 during the first, second, and third wave of this disease and who attended the outpatient cardiology or internal medicine services of our hospital, for persisting symptoms such as long-lasting fatigue, dyspnea, chest pain/discomfort, palpitations, and reduced exercise capacity, we selected a group of 383 previously apparently healthy adults, aged under 55 years, who were infected with the SARS-CoV2 virus 4 to 8 weeks earlier. They all were classified as suffering from a post-acute COVID-19 syndrome and, after excluding residual pulmonary lesions, they were proposed to undergo a comprehensive cardiologic examination, including electrocardiogram (ECG) and TTE. Of them, 281 subjects had normal results, but in 102 patients (26.63%), various pathological TTE findings, such as altered LVF, DD, RVD, elevated sPAP, PE, or PT either isolated, but mostly associated, were identified [12,13]. It is important to mention that all of them had mild/moderate pulmonary injury during the acute illness, documented by chest computed tomography (CCT), performed either during admission in the hospital or at an initial COVID-19 evaluation together with laboratory tests, but none of them were diagnosed with CV complications at that moment. These patients were invited to take part in a follow-up program to assess the progression of these abnormalities at 3 and 6 months (Figure 1).

- Inclusion criteria were:
  1. Apparently healthy patients without a history of CV disease and aged between 18 and 55 years to minimize the possibility of age-induced CV alteration;
  2. The presence of a SARS-CoV2 infection, certified by a positive result of real-time reverse transcriptase-polymerase chain reaction (RT-PCR) assay of nasal and pharyngeal swabs within 4–8 weeks before the first cardiologic examination;
  3. The availability of a discharge summary or an initial COVID-19 assessment with CCT and laboratory tests, documenting the presence of a mild/moderate viral pneumonia during the acute phase, where pulmonary injury under 30% was considered mild and between 30% and 60% was considered of moderate severity;
  4. An initial diagnosis of post-acute COVID-19 syndrome, established based on some long-lasting symptoms such as exhaustion, tiredness, breathing difficulties/dyspnea, palpitations, chest discomfort or angina, reduced effort capacity;
  5. A previous assessment, by TTE, at 4–8 weeks after the acute phase of COVID-19, revealing alterations of cardiac performance, associated or not with elevated sPAP values, and/or the presence of pericardial exudate or thickened pericardium and the patients’ agreement to undergo further cardiologic controls with TTE at 3 and 6 months after the COVID-19 episode.
- Exclusion criteria:
  1. Individuals not capable or not willing to sign an informed consent, as well as those aged under 18 years;
  2. Patients already diagnosed with significant pre-existing cardiovascular diseases or other chronic pathologies, including those aged over 55 years who could have age-related abnormalities;
  3. Subjects not able to provide a baseline COVID-19 evaluation, with CCT describing the severity of the pulmonary injury and laboratory tests;
  4. Patients who suffered from severe/critical forms of COVID-19, with severe respiratory insufficiency, or CV complications requiring intensive care unit hospitalization;
  5. Subjects diagnosed during the study with significant previously unknown cardiac pathology and/or those not willing to undergo all assessments required by the study protocol.

### 2.2. Methods

All subjects who agreed to take part in this study were asked to bring all documents from the acute illness and were scheduled for the next controls after signing an informed consent. We collected their baseline data, their initial CCT, and the control confirming the resolution of the lung injuries and the laboratory results (especially C-reactive protein (CRP) was noted). Their functional status was assessed according to the number of persisting symptoms by using the Post-COVID-19 Functional Status (PCFS) scale, a methodology that estimates the functional limitations during the recovery from COVID-19. In this scale 0 means “no limitations and symptoms”; 1 “negligible limitations of usual activities with persistent symptoms”, 2“slight limitations with significant symptoms”, 3“moderate limitations and not able to perform all usual activities due to symptoms, but still able to take care of himself without assistance” and 4 means “severe limitation due to symptoms and requiring assistance to take care of themselves” [14].

Electrocardiography was performed at the first evaluation and at the subsequent controls, respectively at 3 and 6 months.

Echocardiographic examination was performed at the first evaluation and after 3 and 6 months since the COVID-19 acute illness; comprehensive TTE exams were conducted in all subjects, all TTE measurements being performed according to guidelines recommendations [15]. After a regular exam of the morphology and function of all cardiac chambers, special attention was given to the following evaluations:

- The assessment of LV function (LVF) was realized from apical 4-chamber view and included: (a) the assessment of the LV ejection fraction (LVEF) according to the modified Simpson rule, with values under 50% being considered abnormal; (b) the measurement of the lateral mitral annular plane systolic excursion (MAPSE), normal values being over 10 mm, lower values being pathological; (c) from apical 2-, 3-, and 4-chamber view, we employed strain techniques to evaluate the LV global longitudinal strain (LV-GLS), the region of interest (ROI) being automatically generated, with manual corrections if needed, to adjust the thickness of the LV myocardial wall after tracing the LV endocardial border [16,17]. Values under −18% strongly indicated an impaired LV systolic function.
- The evaluation of LV diastolic dysfunction (DD) included: (a) LV mass index (LVMI), determined from parasternal long axes, with LV hypertrophy (LVH) being confirmed by values of over 115 g/m^2^ for males and 95 g/m^2^ for females; (b) left atrial volume index (LAVI) was assessed from apical 4 chambers view, and values higher than 34 mL/m^2^ were considered abnormal; (c) from the same view, at the level of the mitral valve, we used pulsed Doppler to register the mitral inflow and measured the peak early diastolic velocity (E), the late diastolic velocity (A), and the E/A ratio; (d) at the level of the septal and lateral mitral annulus, tissue Doppler imaging (TDI) was employed to record the early diastolic velocity (e’) and the late diastolic velocity (a’), and an average and E/e’ ratio were calculated. DD of type I was considered if E/A ratio ≤ 0.8 and E < 50 cm/s, and type III DD was certified by an E/A ratio of over 2. In the situation of an E/A ratio ≤ 0.8, with E ˃ 50 cm/sec, or in case of an E/A between 0.8 and 2, DD of type II was assumed, being confirmed by at least two of the following three criteria: an average E/e’ > 14, LAVI > 34 mL/m^2^, and/or TRV > 2.8 m/s. If only one of the three previously mentioned criteria were present, DD of type I was considered [18].
- Right ventricular (RV) function (RVF) was assessed from 4-chamber view and comprised: (a) the measurement, in M-Mode, at the level of the lateral tricuspid valve annulus, of the tricuspid annular plane systolic excursion (TAPSE), with values under 17 mm being pathological; (b) the fractional area change (FAC), from apical view, with levels under 35% being significant for RV dysfunction (RVD); (c) from the same view, by strain techniques, we determined the RV global longitudinal strain (RV-GLS) [19,20], RVD being certified by values < −28%; (d) the estimated systolic PAP (sPAP) was determined based on the peak tricuspid regurgitation velocity (TRV) recorded by continuous Doppler and considering the right atrial pressure, appreciated by measuring the inferior vena cava diameter and its respiratory variations. In our research, we considered that sPAP values of ≥35 mmHg at rest indicate PH [14,16], with severity ranging from mild (35–44 mmHg) to moderate (45–60 mmHg) to severe (>60 mmHg) [17,19];
- The thickness of the pericardial exudate (PE), and/or of the thickened posterior pericardium (PT) were measured from standard views [21].

Statistical analysis:For the statistical analysis of our data, we employed the Statistical Package for the Social Sciences v.26 (SPSS, Chicago, IL, USA). Continuous variables are presented as a mean with standard deviation (SD) or as a median with interquartile range (IQR), and categorical variables as frequency and percentages. Considering that the results of the normality test (Shapiro–Wilk) showed a non-Gaussian distribution, we used further nonparametric tests for our analysis. Spearman’s correlation was employed to determine the potential associations between LV-GLS and E/e’ ratio with other TTE parameters and laboratory results. Friedman’s test followed by a post hoc analysis with Wilcoxon signed-rank test were conducted with a Bonferroni correction to evaluate the evolution of LV-GLS, RV-GLS, FAC, EF, pericardium thickness, and exudate over a follow-up period of 6 months. Several multivariate regression models were built by using the Akaike criterion to assess the impact of several confounding factors on the variance of continuous variables, and the model was validated based on the accuracy of prediction and R squared. In the final regression equations, the predictors were accepted according to a repeated backward-stepwise algorithm (inclusion criteria *p* < 0.05, exclusion criteria *p* > 0.10) to obtain the most appropriate theoretical model to fit the collected data. All *p* values under 0.05 were considered to indicate statistically significant differences.

The Local Scientific Research Ethics Committee of our hospital approved the design 164 and the methodology of our study (No. 206/7.09.2020).

## 3. Results

Out of 383 subjects recovering from COVID-19 who were examined for persisting symptoms, we identified in 102 (26.63%) of them, by TTE, several echocardiographic abnormalities suggesting various CV complications. Our patients were aged between 30 and 55 years, with a mean age of 48.44 ± 5.17 years. A total of 47 of them were men, and 55 were women. Although they were apparently healthy, with no history of CV diseases, an increased percentage of them (38.23%) were overweight or even more (44.11%) were obese, the median body mass index (BMI) being 29.39 (26.58–31.51). Twenty-four of them had blood pressure (BP) values at the upper limit of the normal, with a median systolic blood pressure value of 130 (120–130) mmHg and a diastolic one of 80 (70–80) mmHg. During the acute SARS-CoV-2 infection, all of them had pulmonary injury, ranging from mild (˂30%) in 47 subjects to moderate (30–60%) in 55 individuals. After a cardiologic examination, ECG was performed but, excepting sinus tachycardia, non-characteristic ST segment/T wave changes, or isolated supraventricular/ventricular beats, no peculiar findings were detected. Following a comprehensive TTE assessment, we identified various abnormalities, either as an isolated finding, but more frequently, overlapping one other. We classified our patients, according to the type and severity of the prevailing dysfunction, into four subsets: Group A—35 subjects with impaired LVSF, all of them having also other associated dysfunctions; Group B—51 subjects with elevated sPAP levels, associated or not with RVD and/or reduced LVEF; Group C—66 patients with DD, but with normal LVF (even if borderline); and Group D—23 individuals with pericardial pathology, frequently associated with other dysfunctions. Their clinical and laboratory characteristics during the acute illness and at the first evaluation are presented in Table 1, and the results of the first TTE examination are in Table 2.

Based on the TTE assessment, we determined in the 35 patients (9.12%) from group A with altered LV performance. Among them, 22 were men, and 13 were women, with a mean age of 50.2 (±4.89) years. These patients were older, male gender prevailed, and generally, they had a more severe pulmonary injury during the acute illness and more persisting symptoms during recovery. All of them had reduced LVEF and LV-GLS, and 33 also had reduced MAPSE. Their LVEF varied between 30% and 47%, median 42% (39–44%); LV-GLS values were between −18% and −7%, median −13 % (−15–−11%); and MAPSE was between 5 and 10 mm, median 8 (7–8) mm. All of them also had associated elevated sPAP levels and RVD, with reduced TAPSE, FAC, and RV-GLS (Table 2). DD was also identified in all these subjects, 18 being classified as having type 2 and 17 as type III, with an E/A ratio over 2. By analyzing our results with the Spearman’s correlation, we identified statistically significant correlations between LV-GLS values and other TTE parameters characterizing LVF, sPAP, DD, and RVF (*p* < 0.001), but also with the initial levels of the lung injury and CRP, weeks elapsed since COVID-19, and the PCFS scale (*p* < 0.001) (Table 3). They were treated with beta-blockers, angiotensin-converting enzyme inhibitors, and diuretics if needed.

In group B, consisting of 51 patients, increased sPAP values, associated or not with RVD, expressed by reduced levels of TAPSE, FAC, and/or RV-GLS, were identified. All these subjects had associated DD: 4 of them had type I, 27 had type II, and 20 had type III. Thirty-five of them also had reduced LVF.

In group C were included 66 patients with normal systolic function, and various patterns of DD were diagnosed as follows: 26 subjects had E/A ratio ≤ 0.8 and E < 50cm/s and were classified as DD type I, and in 5 cases with E/A ratio ˃ 2 DD, type III was confirmed. In the remaining 35 subjects with an E/A ratio ≤ 0.8 but with an E of over 50 cm/s or with the E/A ratio between 0.8 and 2, DD of type II was confirmed based on at least two of the following criteria: an average E/e’ > 14, LAVI > 34 mL/m^2^ and/or TRV > 2.8 m/s. The remaining 16 who fulfilled only one criterion were reclassified as DD type I, resulting in a final number of 42 subjects. However, these patients were younger, female gender prevailed, 22 had obesity grade I, 1 had grade II, and 28 were overweight, and in 36 patients, 11 men and 25 women, LVH was evidenced. By analyzing these results with the Spearman’s correlation, we evidenced statistically significant correlations between E/e’ ratio and various other parameters such as LV-GLS, LVEF, MAPSE, sPAP, RV-GLS, and PT (*p* < 0.001) and with the initial CCT score and CRP levels, (*p* < 0.001), as well as with age, BMI, and PCSF scale, all with *p* < 0.001 (Table 4).

Another peculiar finding on TTE was the presence of a small pericardial effusion associated or not with a thickened pericardium, which was found in 19 subjects, while another 4 had only thickened pericardium. Most patients had also reduced LVEF (21 subjects), 2 of them having borderline values (LVEF = 50%).

**Figure 2:**
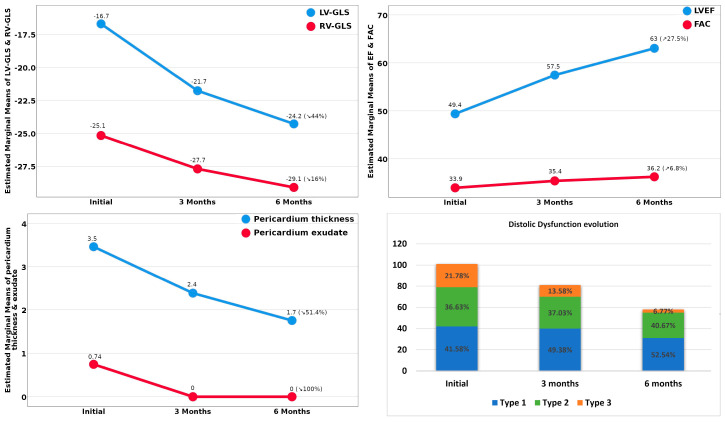
Evolution of the TTE-assessed abnormalities identified in study patients.

As we followed the evolution of these TTE-determined abnormalities, we noticed a gradual, significant improvement in all those parameters at 3 months, but especially after 6 months. In group A, the TTE parameters characterizing LVF returned to normal in 94.28% of patients, the median values of LV-GLS improved from −13%(−15% to −11%) to −23%(−24% to −22%), parallel with LVEF and MAPSE. At the end of the follow-up, only 5.7% still had moderately reduced LVEF (between 40% and 49%), but it is important to mention that values at the lower normal limit, often associated with DD, were still found in one-third of these patients (Figure 2). We built a regression model, and after the adjustment of confounding factors, we documented that 62.1% of the LV-GLS levels at 6 months, and of LVEF as well, were related to more advanced age, the initial values of CRP and lung injury, and to the baseline sPAP values, but also to some differences in the pathogeny of the viral strains involved in the three infection waves (R^2^ = 0.621), the latest (the VOC 202012/01 strain) being associated with a worse evolution. In group B, we noticed a gradual improvement in RVD parallel with the reduction in sPAP levels, but at the end of the follow-up, there were still several patients (15.6%) with slightly pathological values. As we analyzed the evolution of DD in group C, we observed that although at the end of the follow up almost half of the patients still had some abnormalities characterizing DD, its severity decreased so that after 6 months only 6.77% of the patients had type III of DD, in comparison with 21.78% at the beginning of our study (Figure 2).

In order to assess the independent factors that predict the E/e’ ratios’ values at 6 months, we employed a multivariate linear regression. As it is known that DD is influenced by several factors, we included in our model age, gender, BMI, BP, pericardium thickness, effusion, PCFS, CRP, and other parameters related to the COVID-19 infection, such as the extent of the pulmonary injury and the weeks since infection. The model explained 30.5% of the E/e’ variance (adjusted R^2^ = 0.305), and it is worth mentioning that the E/e’ ratio decrease at 6 months was related not only to the younger age of the patient (as expected) but also to a lower CRP level during the acute infection and mostly by the various strains responsible for the SARS-CoV-2 infection—the patients infected with the newest strains/wave 3 had higher E/e’ values at 6 months. The existence and degree of the initial pericardium exudate/thickening also increased the 6 months E/e’ ratio, but the level of significance was only marginal.

Referring to group D, the evolution was favorable in all patients; PE disappeared completely after 3 months, and PT decreased under 4 mm at 6 months. Factors that seemed to influence this evolution were the patient’s age and the presence of associated LV systolic dysfunction and/or of RVD (R^2^ = 0.742) (Figure 1).

## 4. Discussion

Although it is well established that in COVID-19 infection the respiratory manifestations dominate the clinical picture, culminating in the dramatic image of respiratory insufficiency, which represents the principal cause of death, it is now well established that the evolution of this disease is even worse in patients with associated CV pathology—either preexisting or newly developed during COVID-19. If the acute CV manifestations of this disease are largely debated in the medical literature [1,6,8,22], more concern has raised the assumption that there could be long-term consequences, explaining, at least partially, the long-lasting symptomatology during the recovery from the infection with the SARS-CoV-2 virus, or the so-called post-acute or long COVID-19 syndrome [9,23,24]. Another possibility is that in many cases, where the cardiac complaints were not so significant during the acute illness, the CV complications could have been overlooked, and patients could have returned to a physician due to persisting symptoms, being diagnosed in the sub-acute phase [9,25]. However, another aspect of this concern is that there are now studies that debate chronic cardiac complications after COVID-19 when the viral load is already normalized [6,26,27,28,29].

In our study, we debated four principal patterns of CV abnormalities assessed by TTE in 102 patients during the recovery from COVID-19 at 4 to 8 weeks after the acute illness. None of these patients was diagnosed during the acute SARS-CoV-2 infection with CV complications and neither did they have a previous history of CV disease. A first pattern, diagnosed in one-third of our patients, mostly men, generally with more severe lung injury during the acute infection, was that of impaired LV systolic function, confirmed by lower LV-GLS and associated with reduced LVEF and MAPSE. All of them also had other TTE abnormalities such as DD and/or elevated sPAP. These alterations could represent the consequence of a myocardial injury that occurred either during the acute illness or in the course of recovery, being mediated by immune mechanisms [26,30]. Acute myocarditis and heart failure following a SARS-CoV-2 infection were frequently described in the medical literature [3,31,32], with some of them with fulminant evolution but with most of them alleviating in time [2,33]. In our follow-up, at 6 months, we still evidenced in 5.7% of patients’ mild alterations of the TTE parameters characterizing LVF. DD was the most frequent pattern assessed in our patients, also identified as an isolated dysfunction (with normal LVEF), mostly in female patients, many of them overweight, who had a less severe lung injury during the acute illness. It is also possible that several patients that had blood pressure values at the upper normal values could have had subclinical, not previously diagnosed, LVH that could explain, at least partially the presence of DD. Thus, it is important to mention that from this group, at the end of the follow-up, some alterations defining DD persisted in one-third of the patients, raising the presumption of remaining interstitial myocardial changes, potentially evolving to ICF, which is an aspect also debated in other studies [5,10,23,28,31]. Considering that the TTE diagnosis of DD represents a combination of three, two, or one elements from several abnormalities, some of them, such as TRV, being influenced also by the pulmonary pathology, it is still important to mention that the follow up of its evolution is challenging.

As COVID-19 is a new disease associated with multiple organ damage, the evolution of the acute and especially of the chronic cardiac complications following this disease is still a matter of debate. It is assumed that most of the cases have a favorable course over time, but exact data are lacking. Another still unknown aspect is the relationship between the pathogeny of the viral strain and the severity, evolution, and long-term outcome of the cardiac dysfunction following this infection. Many studies and meta-analyses are needed to clarify these aspects [34].

One of the most important limitations of this study is that we did not have a detailed TTE exam performed during the acute phase of the SARS-CoV-2 infection, either in the inpatients or in the outpatients as well. Furthermore, we assumed that our patients had no cardiovascular diseases prior to the illness because they had a previous echocardiographic exam performed sometime, but it was in most cases an abbreviated exam, recording only “within normal limits” or “without significant pathological findings”, without accurate measurements. Another limitation was that we could not compare our TTE findings with the results of other imagistic examinations, especially with magnetic resonance imaging [35], due to the delay caused by the scheduling of this procedure considering the difficulties raised by the pandemic.

## 5. Conclusions

Multiple cardiac abnormalities can be diagnosed by transthoracic echocardiography in patients recovering from COVID-19, even in those not diagnosed during the acute infection with cardiovascular complications. Certain of these alterations persist several weeks or even months after the acute illness, explaining some of the persistent symptoms included in the post-acute COVID syndrome. They seem to alleviate gradually, parallel with the months elapsed since the acute illness, apparently not only in relation to patients’ age and the severity of the acute infection but also with the pathogeny of different viral strains. 

## Figures and Tables

**Table 1:** Clinical and laboratory data of study population.

Patients’ Characteristics	Group A 35 Patients	Group B 51 Patients	Group C 66 Patients	Group D 23 Patients
Age (years)	50.2 (±4.89)	49.29 (±5.74)	47.63 (±5.06)	48.78 (±5.41)
Gender: male	22 (62.85%)	28 (54.90%)	25 (37.87%)	15 (65.21%)
female	13 (37.14%)	23 (45.09%)	41 (62.12%)	8 (34.78%)
Initial pulmonary injury assessed on CCT	38 (35–40)	35 (31–40)	23.5 (14.75–30)	40 (35–40)
Mild:˂30%	3 (8.57%)	8 (15.68%)	43 (65.15%)	3 (13.04%)
Moderate: 30–60%	32 (91.42%)	43 (84.31%)	23 (34.84%)	20 (86.95%)
Initial CRP (mg/dL)	45.6 (40.1–56.3)	42.5(39.1–50.8)	35.7 (28.5–39.7)	47.9 (42.1–57.9)
Clinical findings at the inclusion in the study				
BMI (Kg/m^2^)	31.1 (27.7–32.9)	30.5 (27.5–31.8)	27.6 (25.6–30.5)	30.5 (27.1–31.7)
Heart rate (b/min)	85 (80–90)	80 (80–86)	75 (75–80)	85 (80–90)
Blood pressure (mmHg)				
Systolic	135 (130–140)	130 (130–140)	130 (120–130)	130 (130–140)
Diastolic	80 (80–90)	80 (70–85)	70 (70–80)	80 (75–90)
PCFS scale	3 (3–3)	3 (2–3)	2 (2–2)	3 (3–3)
Weeks since COVID-19	5 (4–6)	6 (4–7)	8 (7–8)	5 (4–6)
Electrocardiography				
Sinus tachycardia (˃ 80 b/min)	18 (51.42%)	25 (49.01%)	13 (19.69%)	12 (52.17%)
Non-specific ST/T changes	13 (37.14%)	15 (29.41%)	11 (16.66%)	11 (47.82%)
Isolated PSVB/PVB	17 (48.57%)	17 (33.33%)	13 (19.69%)	7 (30.43%)

Legend: Group A—35 subjects with impaired LVSF; Group B—66 patients with diastolic dysfunction but with normal LV systolic performance; Group C—51 subjects with elevated sPAP levels, associated or not with RVD; Group D—23 individuals with pericardial pathology; CCT—chest computed tomography; CRP—C-reactive protein; BMI—body mass index; PCFS—Post-COVID-19 Functional Status scale; PSVB—premature supraventricular beats; PVB—premature ventricular beats.

**Table 2:** Echocardiographic findings at the first evaluation.

TTE Results at the First Evaluation	Group A	Group B	Group C	Group D
LVMI (g/m^2^)	110 (94.56–118)	98.13 (94–116.73)	98.69(94.48–112.83)	109.12 (96.7–117.65)
LAVI (mL/m^2^)	30.45 (22.9–35.4)	29.7 (21.59–34.85)	29.51 (20.12–34.05)	30.45 (22.9–35.4)
Pericardial exudate (mm)	4 (3.67–4.15)	4 (3.6–4.1)	3.2 in 1 patient	4 (3.6–4.1)
Pericardial thickness	4.3 (3.6–6)	3.8 (3.4–5.2)	2.85 (2.15–3.52)	5.6 (4.3–6)
MAPSE (mm)	8 (7–8)	8 (7–10)	12 (11–14.25)	7 (7–8)
LVEF (%)	42 (39–44)	43 (40–50)	54.5 (50–56.25)	41 (38–43)
LV-GLS (%)	−13 (−15–−11)	−15 (−17–−12)	−19 (−21–−18)	−13 (−15–−11)
TAPSE (mm)	15.3 (13–16)	16 (15–17)	19 (18–20.25)	15.3 (13–16)
FAC (%)	31.78 (30–33.56)	33.11(30.24–34.02)	35.29 (34.5–35.91)	31.23 (29–33.56)
RV-GLS (%)	−20(−24–−19)	−22 (−25–−19)	−28 (−29–−27)	−20 (−22–−19)
TRV (m/sec)	3.23 (3.12–3.35)	3.15 (2.98–3.3)	2.7 (2.68–2.74)	3.28 (3.17–3.39)
sPAP (mmHg)	46.73 (43.93–49.9)	44.68 (40.52–40.56)	34.16 (33.72–34.98)	48.03 (45.19–50.96)
E/A	2.03 (0.96–2.11)	1.4 (0.94–2.1)	0.94 (0.74–1.26)	2.05 (1.27–2.12)
E/e’	14.44 (14.18–15.1)	14.35 (14.12–14.79)	14.13 (13.38–14.32)	14.52 (14.15–15.0.9)

Legend: Group A—35 subjects with impaired LVSF; Group B—66 patients with diastolic dysfunction but with normal LV systolic performance; Group C—51 subjects with elevated sPAP levels, associated or not with RVD; Group D—23 individuals with pericardial pathology; LVMI—left ventricular mass index; LAVI—left atrial volume index; MAPSE—mitral annular plane systolic excursion; LVEF—left ventricular ejection fraction; LV-GLS-left ventricular global longitudinal strain; TAPSE—tricuspid annular plane systolic excursion; FAC—fractional area change; RV—GLS-right ventricular global longitudinal strain; TRV—peak tricuspid regurgitation velocity; sPAP—systolic pressure in the pulmonary artery; E/A—peak mitral inflow early (E) to late (A) diastolic velocities in pulsed Doppler; E/e’—early mitral inflow diastolic velocity E to average e’ velocity (E/e’) in pulsed tissue Doppler.

**Table 3:** Correlations between LV-GLS, other TTE parameters, and laboratory data in study patients.

	Age	LVEF	MAPSE	sPAP	RV-GLS	E/e’	Weeks	CCT Score	CRP	PCFS Scale
**R**	0.248	−0.851	−0.745	0.699	0.678	0.433	−0.588	0.525	0.522	0.529
**95%CI**	0.055	−0.919	−0.825	0.560	0.534	0.231	−0.708	0.369	0.343	0.365
	0.434	−0.747	−0.627	0.800	0.784	0.609	−0.441	0.646	0.674	0.674
***p***	0.012	<0.001	<0.001	<0.001	<0.001	<0.001	<0.001	<0.001	<0.001	<0.001

Legend: LV-GLS—left ventricular global longitudinal strain; TTE—transthoracic echocardiography; LVEF—left ventricular ejection fraction; MAPSE—mitral annular plane systolic excursion; sPAP—systolic pressure in the pulmonary artery; RV-GLS—right ventricular global longitudinal strain; E/e’—early mitral inflow diastolic velocity E to average e’ velocity in pulsed tissue Doppler; CCT—chest computed tomography; CRP—C-reactive protein; PCFS—Post-COVID-19 Functional Status scale; Spearman correlation test.

**Table 4:** Correlations between E/e’ other TTE parameters and laboratory data in study patients.

	Age	LVEF	MAPSE	sPAP	RV-GLS	BMI	PT	CCT Score	CRP	PCFS Scale
**R**	0.358	−0.499	−0.426	0.454	0.474	0.222	0.423	0.376	0.547	0.364
**95%CI**	0.172	−0.603	−0.603	0.249	0.293	0.028	0.225	0.180	0.353	0.144
	0.520	−0.311	−0.213	0.633	0.638	0.401	0.589	0.573	0.699	0.539
***p***	<0.001	<0.001	<0.001	<0.001	<0.001	0.025	<0.001	<0.001	<0.001	<0.001

Legend: E/e’—early mitral inflow diastolic velocity E to average e’ velocity in pulsed tissue Doppler; transthoracic echocardiography; LVEF—left ventricular ejection fraction; MAPSE—mitral annular plane systolic excursion; sPAP—systolic pressure in the pulmonary artery; RV-GLS—right ventricular global longitudinal strain; BMI—body mass index; PT—pericardial thickness; CCT—chest computed tomography; CRP—C-reactive protein; PCFS—Post-COVID-19 Functional Status scale; Spearman correlation test.

## Data Availability

Our data are available on https://doi.org/10.17632/yw4jhs5n9d.1 (accessed on 1 January 2022).
